# Theobromine Does Not Affect Fasting and Postprandial HDL Cholesterol Efflux Capacity, While It Decreases Fasting miR‐92a Levels in Humans

**DOI:** 10.1002/mnfr.201800027

**Published:** 2018-06-19

**Authors:** Charlotte P.J. Talbot, Ronald P. Mensink, Lotte Smolders, Virginie Bakeroot, Jogchum Plat

**Affiliations:** ^1^ Department of Nutrition and Movement Sciences NUTRIM School of Nutrition and Translational Research in Metabolism Maastricht University Universiteitssingel 50 6229 ER Maastricht The Netherlands

**Keywords:** cholesterol efflux capacity, high‐density lipoprotein, microRNAs, postprandial, theobromine

## Abstract

**Scope:**

Chocolate consumption lowers cardiovascular disease risk, which might be attributed to the methylxanthine theobromine. These effects may be mediated through effects on HDL‐mediated cholesterol efflux, which may be affected by microRNA (miRNA) levels in the HDL particles. Therefore, the aim of this study is to investigate effects of theobromine consumption on fasting and postprandial cholesterol efflux and miRNAs levels.

**Methods and results:**

Thirty overweight and 14 obese healthy men and women participated in this randomized, double‐blind crossover study. Participants consumed 500 mg d^−1^ of theobromine or placebo for 4 weeks. ABCA1‐mediated cholesterol efflux was measured using J774 macrophages. MiRNAs levels (miR‐92a, miR‐223, miR‐135a*) were quantified in apolipoprotein B‐depleted serum. Theobromine consumption did not affect fasting and postprandial cholesterol efflux. Fasting miR‐223 and miR‐135a levels were unchanged, while miR‐92a levels were decreased (−0.21; *p* < 0.05). The high‐fat meal increased postprandial cholesterol efflux capacity (+4.3 percentage points; *p* ≤ 0.001), miR‐92a (+1.21; *p* < 0.001), and miR‐223 (+1.79; *p* < 0.001) levels, while a trend was found for miR‐135a (+1.08; *p* = 0.06).

**Conclusion:**

Theobromine did not improve fasting and postprandial ABCA1‐mediated cholesterol efflux capacity, but decreased fasting miR‐92a levels. High‐fat meal intake increased postprandial cholesterol efflux and the three selected miRNAs levels.

## 1 Introduction

MicroRNAs (miRNA) are small, non‐coding RNAs composed of about 22 nucleotides that bind to complementary sites in the 3’UTR region of their target RNAs, thereby inhibiting gene expression through posttranscriptional regulation.^1^ MiRNAs can be encapsulated within vesicles, such as exosomes, which are subsequently released into the circulation. However, miRNAs can also bind to protein complexes or be associated with lipoproteins.^2^ Vickers et al. were the first to demonstrate that high‐density lipoprotein (HDL) particles isolated from human plasma contain miRNAs, which can subsequently be transmitted in vitro to hepatocytes cells via scavenger receptor BI (SR‐BI).^3^ In addition, HDL miR‐223 can be transferred from HDL particles to human coronary artery endothelial cells, also through SR‐BI.^4^ MiRNAs are also transported by other lipoproteins, but in smaller amounts.^5^

In humans, the HDL‐miRNAs signature is different under cardiovascular and hypercholesterolemic conditions, although the specific roles of the different miRNAs are largely unknown.^3^ Among the many miRNAs, miR‐223, miR‐135a, and miR‐92a belong to the most abundant HDL‐associated miRNAs.^3^, ^5^, ^6^ MiR‐223 has previously been reported to indirectly increase cholesterol efflux, the main anti‐atherogenic effect of HDL.^7^ Whether miR‐135a and miR‐92a are also related to cholesterol efflux has never been studied.

Not much is known about the effects of diet on HDL‐mediated cholesterol efflux and miRNAs concentrations, which may provide explanations for observed associations between diet or dietary components with CVD risk. Chocolate consumption, for example, is associated with a reduction in the risk to develop cardiovascular disease (CVD).^8^ These effects may be mediated through effects on flow‐mediated vasodilation, which has been reported to increase after dark chocolate intake.^9^ Also, HDL‐C concentrations may increase, although results are controversial.^10^, ^11^, ^12^ However, it is questionable whether increasing HDL‐C concentrations protects against CVD.^13^ Further, it has been suggested that only dark chocolate have cardioprotective effects. Dark chocolate is rich in methylxanthines, including theobromine,^14^ which may increase HDL‐C and apoA‐I concentrations, the main apolipoprotein within HDL.^15^ However, effects of theobromine consumption on fasting and postprandial HDL‐mediated cholesterol efflux capacity have never been investigated. In fact, not much is known at all about changes in cholesterol efflux during the postprandial phase, although disturbances in postprandial lipoprotein metabolism are an important risk factor for CVD.^16^, ^17^ Therefore, the effect of theobromine intake on HDL‐mediated cholesterol efflux capacity was investigated in 44 overweight and obese men and women with low HDL‐C concentrations. Since an increased BMI and low HDL‐C concentrations are associated with a decreased ABCA1‐mediated cholesterol efflux,^18^ it was hypothesized that cholesterol efflux capacity improves after theobromine intake, which could therefore contribute to the potential beneficial effect of dark chocolate consumption on CVD risk. In addition, changes in miRNAs levels in apolipoprotein B (apoB)‐depleted serum (e.g., miR‐223, miR‐135a*, and miR‐92a) following theobromine and meal consumption were examined. Finally, relationships between changes in the expression of these miRNAs and variations in cholesterol efflux capacity as well as serum lipids were assessed.

## 2 Experimental Section

### 2.1 Subjects

Thirty overweight (body mass index, BMI, kg m^−2^: 25–30) and 14 obese (BMI: 30–35) apparently healthy subjects participated in this study, which examined the effects of theobromine consumption on cardiometabolic health. The 28 men were aged 45–70 years (60.2 ± 5.2 years), with a BMI of 28.6 ± 2.7. Six of the men were obese. The 16 women were aged 50–70 years (60.5 ± 6.0 years) with a BMI of 30.2 ± 3.2. Eight of the women were obese. All subjects had low baseline HDL‐C concentrations (<1.2 mmol L^−1^ for men and <1.5 mmol L^−1^ for women) and were not using any medications. Baseline characteristics of the study population are presented in the Table S1, Supporting Information. The study was conducted according the ethical guidelines of the 1975 Declaration of Helsinki and approved by the Ethics Committee of Maastricht University Medical Centre. Written informed consent was obtained from all participants before entering the study. The study was registered at clinicaltrials.gov as NCT02209025.

### 2.2 Studies Design

This study had a randomized, double‐blinded placebo‐controlled, crossover design, including two intervention periods of 4 weeks, which were separated by a 4‐week washout period. Two weeks before the start of the study and during the study, subjects had to avoid the use of products containing cocoa, as well as to limit their consumption of caffeine‐containing drinks, as theobromine is a metabolite of caffeine. Subjects were randomly allocated to a group starting with a theobromine or placebo drink. During the 4 weeks intervention periods, subjects had to consume daily a 20 mL drink enriched with either 500 mg theobromine or placebo. At the end of both 4 weeks intervention periods (day 28), subjects participated in a postprandial test, for which they had to consume a shake (460 mL) that provided 965 kcal (17.9 g proteins, 85.7 g carbohydrates, and 60.6 g fat), which had to be consumed within 10 min together with their test drink (Table S2, Supporting Information).

### 2.3 Blood Sampling

At day 28 of both the intervention and the placebo period, an intravenous cannula was inserted into a forearm vein and fasting blood samples were collected before the intake of the shake (*T* = 0). Postprandial blood samples were sampled 2 h (*T* = 120) following shake consumption. Blood was collected in a 4 mL EDTA tube and then centrifuged at 1300 × *g* for 15 min at 4 °C to obtain plasma. To obtain serum, samples were allowed to clot for 1 h at 20 °C, and then centrifuged at 1300 × *g* for 15 min at 20 °C. Plasma and serum supernatants were transferred into 1.5 mL Eppendorf tubes and stored at −80 °C until use.

### 2.4 Biochemical Measurements

In all fasting (*T* = 0) and postprandial (*T* = 120) serum samples obtained at day 28 of both periods, HDL‐C concentrations (precipitation of apoB‐containing lipoproteins; Roche Diagnostics System, Hofmann‐La Roche Ltd., Basel, Swiss), apoA1 (immunoturbidimetric reaction; Horiba ABX, Montpellier Cedex, France), and triacylglycerol (TAG) (TRIGL: ACN 8781; Roche, Mannheim, Germany) concentrations were determined. All samples from one subject were analyzed within the same run. As only values at day 28 were used in the present study, results may therefore slightly differ from a previous study, which reported the average values.^19^

The ability of the HDL particles to promote cholesterol efflux from macrophages was measured as previously described.^20^ Briefly, murine J774 macrophages were cultivated and incubated overnight with BODIPY cholesterol. Afterward, macrophages were equilibrated with an ACAT inhibitor (Acyl CoA Acyltransferase Inhibitor; Sandoz 58‐035) to block cholesterol esterification, and the cholesterol‐efflux transporter ABCA1 was upregulated with cAMP (cyclic adenosine monophosphate; C3912, Sigma). To obtain HDL, human serum samples were depleted of apolipoprotein‐B containing lipoproteins using tungstophosphoric acid hydrate and magnesium chloride, and the isolated HDL particles were then incubated with the labeled macrophages for 4 h. All steps were performed in the presence of 1 μL mL^−1^ of ACAT inhibitor. For normalization, serum samples from two healthy subjects were included in each assay. The efflux capacity values obtained for each subject were normalized to the average efflux capacity value of these two pooled serum, which was set to 100% (% pools). All samples were analyzed in triplicate.

### 2.5 MicroRNAs Analysis

#### 2.5.1 HDL Isolation

To study the potential role of miRNAs on HDL functionality, HDL particles were isolated from EDTA plasma samples by precipitation of apoB‐containing lipoproteins using polyethylene glycol (PEG, molecular weight (MW) 6000; Sigma‐Aldrich, St. Louis, MO), as previously described.^21^ Briefly, 200 μL PEG (200 mg mL^−1^) was added to 500 μL of plasma. Samples were incubated for 20 min at room temperature, and centrifuged at 10 000 rpm for 30 min. Supernatants, containing the HDL particles, were collected in 1.5 mL Eppendorf tubes and stored at −80 °C until use. Since it cannot be excluded that the miRNAs were solely associated with HDL, but also with other carriers present in the apoB‐depleted supernatant (e.g., exosomes), the results are further referred to as miRNAs in the apoB‐depleted plasma fraction.

#### 2.5.2 Total RNA Isolation

Total RNA was isolated from 150 μL isolated HDL, using the miRVana PARIS kit (Ambion, Austin, TX, USA). Samples were eluted with 50 μL preheated at 95 °C elution solution. For normalization of miR expression, samples were spiked with 1.6 μL of 5 nM of *Caenorhabditis elegans* (cel) miR‐39 (cel‐miR‐39, RNA oligonucleotide, Sigma‐Aldrich) after the addition of the denaturing solution. Cel‐miR‐39 was used as external control because of the absence of homologous sequences in humans.^22^

#### 2.5.3 MicroRNA Individual TaqMan Assays

MicroRNAs known to be present in HDL particles and described to be altered in cardiometabolic diseases were chosen, that is, miR‐135a*, miR‐223‐3p, and miR‐92a.^3^, ^5^, ^6^ The primer sequences used are shown in **Table** 1.

**Table 1:** miRNAs primer sequences

miRNA	Primers sequence
hsa‐miR‐135a* (3p)^3^	5’ UAUAGGGAUUGGAGCCGUGGCG 3’
hsa‐miR‐223‐3p^3^	5’ UGUCAGUUUGUCAAAUACCCCA 3’
hsa‐miR‐92a^6^	5’ UAUUGCACUUGUCCCGGCCUGU 3’
cel‐miR‐39^57^	5’ UCACCGGGUGUAAAUCAGCUUG 3’

Total RNA was reverse transcribed using the TaqMan MicroRNA Reverse Transcription Kit (Applied Biosystems, Thermo Fisher Scientific, USA) in a total reaction volume of 15 μL. Briefly, 6 μL of the total RNA including the external control (5 nM) was used in addition with 3 μL of the specific miRNAs primer (5X) and 6 μL of the Mix solution (i.e., 1.5 μL 10X RT buffer, 0.15 μL dNTPs, 0.19 μL RNAse inhibitor, 1 μL Multiscribe RT enzyme and 3.16 μL water). The reaction mixtures were incubated at 16 °C for 30 min, followed by 30 min at 42 °C and 5 min at 85 °C, and then held at 4 °C.

Subsequently, individual TaqMan MicroRNA Assays (Applied Biosystems, USA) were used for detection of miRNAs expression by real‐time PCR. For this, 3 μL of the reverse transcription product was mixed with 10 μL of the Taqman Universal PCR Master Mix II (2X) no AmpErase UNG (Applied Biosystems, USA), together with 1 μL of specific miRNAs primer (20X) and water to a final reaction volume of 20 μL. Real‐time PCR was performed in duplicate on a 7300 real‐time PCR system (Applied Biosystems, USA), and the reaction mixtures were incubated at 95 °C for 10 min, followed by 40 cycles of 95 °C for 15 s and 60 °C for 1 min. The cycle threshold (CT) values were calculated with SDS v1.4 software using automatic threshold (Applied Biosystems, USA). The expression levels of the target miRNAs in apoB‐depleted serum were normalized relative to the expression of cel‐miR‐39, and were calculated using the 2−^ΔΔCT^ method in order to determine the fold change in gene expression.^23^

### 2.6 Statistics

A paired‐samples *t*‐test was used to examine the effects of theobromine on fasting parameters. Postprandial changes were calculated as the difference between 2 h (*T* = 120) after the intake of the mixed meal and fasting measurements (*T* = 0), and were analyzed during the placebo and theobromine period separately with a paired‐samples *t*‐test. Differences in postprandial changes between the two periods were also analyzed with a paired‐samples *t*‐test.

For the miRNAs analysis, Grubbs’ test was used for the detection of outliers that were not physiologically plausible, which were removed from analysis. To examine the postprandial and diet effects on miRNAs levels, a one‐samples *t*‐test was used, with the baseline test value (*T* = 0 or placebo) set to 1. Differences in changes in miRNAs levels between the two periods were performed with a paired‐samples *t*‐test. Correlations between fold changes of miRNA levels with age, as well as with changes in biochemical parameters (cholesterol efflux capacity and concentrations of HDL‐C, apoA‐I, and TAG) after the high‐fat test meal intake were performed using bivariate Pearson correlation coefficients.

A *p*‐value ≤ 0.05 was considered significant. All statistical analyses were performed with SPSS 23.0 (IBM, Armonk, NY) for Mac OS.

## 3 Results

### 3.1 Effects of Theobromine on Fasting and Postprandial Cholesterol Efflux, Serum Lipids, and Lipoproteins

Four weeks theobromine supplementation did not affect fasting cholesterol efflux capacity (+0.4 percentage point (pp); 95% CI: −2.81, 3.57; *p* = 0.81), but significantly increased HDL‐C concentrations (+0.04 mmol L^−1^; 95% CI: 0.003, 0.07; *p* < 0.05). Furthermore, apoA1 (+0.01 g L^−1^; 95% CI: −0.02, 0.05; *p* = 0.50) and TAG concentrations (0.15 mmol L^−1^; 95% CI: −0.03, 0.33; *p* = 0.10) did not change.

During the control period, the intake of the test meal significantly increased cholesterol efflux after 2 h (+4.3 pp; 95% CI: 1.87, 6.68; *p* ≤ 0.001). However, during the theobromine period, no postprandial increase was observed (+1.6 pp; 95% CI: −1.25, 4.42; *p* = 0.26). The test meal did not affect HDL‐C concentrations in both conditions, that is, without (+0.01 mmol L^−1^; 95% CI: −0.01, 0.03; *p* = 0.16) or with theobromine; (+0.003 mmol L^−1^; 95% CI: −0.01, 0.02; *p* = 0.69), During the control period, apoA1 concentrations (+0.02 g L^−1^; 95% CI: 0.004, 0.05; *p* < 0.05) and TAG concentrations (+1.23 mmol L^−1^; 95% CI: −1.04, 1.43; *p* ≤ 0.001) significantly increased after test meal consumption. Comparable results were found during the theobromine period (apoA1: +0.03 g L^−1^; 95% CI: 0.01, 0.04; *p* < 0.001; TAG: +1.23 mmol L^−1^; 95% CI: 1.03, 1.42; *p* < 0.001).

Postprandial changes were similar between both periods for cholesterol efflux (−2.7 pp; 95% CI: −0.657, 1.19; *p* = 0.17), as well as for the concentrations of HDL‐C (−0.01 mmol L^−1^; 95% CI: −0.04, 0.02; *p* = 0.47), apoA1 (0.002 g L^−1^; 95% CI: −0.02, 0.03; *p* = 0.85) and TAG (0.02 mmol L^−1^; 95% CI: −0.15, 0.18; *p* = 0.85) (**Table** 2).

**Table 2:** Effect of 4‐week theobromine supplementation on fasting and postprandial responses in cholesterol efflux capacity, lipids, and apolipoprotein concentrations

	Placebo			Theobromine		
	*T* = 0	*T* = 120	Change *T*_120_–*T*_0_	*T* = 0	*T* = 120	Change *T*_120_–*T*_0_
Cholesterol Efflux [% pools]	97.1 ± 11.5	101.4 ± 11.1	4.3 ± 7.8**	97.5 ± 9.2	99.1 ± 11.7	1.6 ± 9.2
HDL‐C [mmol L^−1^]	1.04 ± 0.19	1.06 ± 0.18	0.01 ± 0.07	1.08 ± 0.22a)	1.09 ± 0.23	0.00 ± 0.06
ApoA1 [g L^−1^]	1.33 ± 0.16	1.36 ± 0.16	0.02 ± 0.07*	1.34 ± 0.18	1.37 ± 0.20	0.03 ± 0.05**
TAG [mmol L^−1^]	1.68 ± 0.72	2.91 ± 1.22	1.23 ± 0.62**	1.83 ± 0.98	3.06 ± 1.46	1.23 ± 0.64**

Values are means ± SD, *n* = 44

Differences between the theobromine and the placebo groups were performed with a paired‐samples *t*‐test: significantly different than placebo at *T* = 0; ^a)^ ≤ 0.05

Postprandial effect was assessed with a paired‐samples *t*‐test: *T* = 120 significantly different from *T* = 0; ^*^ *p* ≤ 0.05; ^**^ *p* ≤ 0.001

Difference in postprandial changes between theobromine and placebo was performed with a paired‐samples *t*‐test.

### 3.2 Effect of Theobromine on Fasting and Postprandial MiRNAs Levels

Using the Grubbs’ test for normality, 17 out of the 396 miRNA measurements carried out in this study were removed from the analysis. For the fasting results, nine values were omitted. For the postprandial results, five values were removed in the placebo period, while three values were removed in the theobromine period.

**Figure 1:**
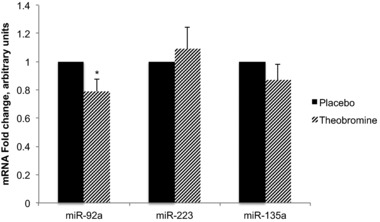
Fold changes of fasting miRNAs levels after 4 weeks of theobromine supplementation. Fasting gene expression after 4 weeks of theobromine consumption relative to fasting gene expression after 4 weeks of placebo consumption. Difference between theobromine and placebo (diet effect at T0) was performed with a one‐samples *t*‐test, with the test value set to 1: *p ≤ 0.05. miR‐92a: *n* = 40. miR‐223: *n* = 41. miR‐135a: *n* = 42.

Theobromine supplementation for 4 weeks reduced fasting miR‐92a levels (−0.21; 95% CI: −0.38, −0.03; *p* < 0.05), while no effects were found on miR‐223 (+0.09; 95% CI: −0.22, 0.41; *p* = 0.55) and miR‐135a levels (−0.12; 95% CI: −0.35, 0.10; *p* = 0.26; **Figure** 1).

**Figure 2:**
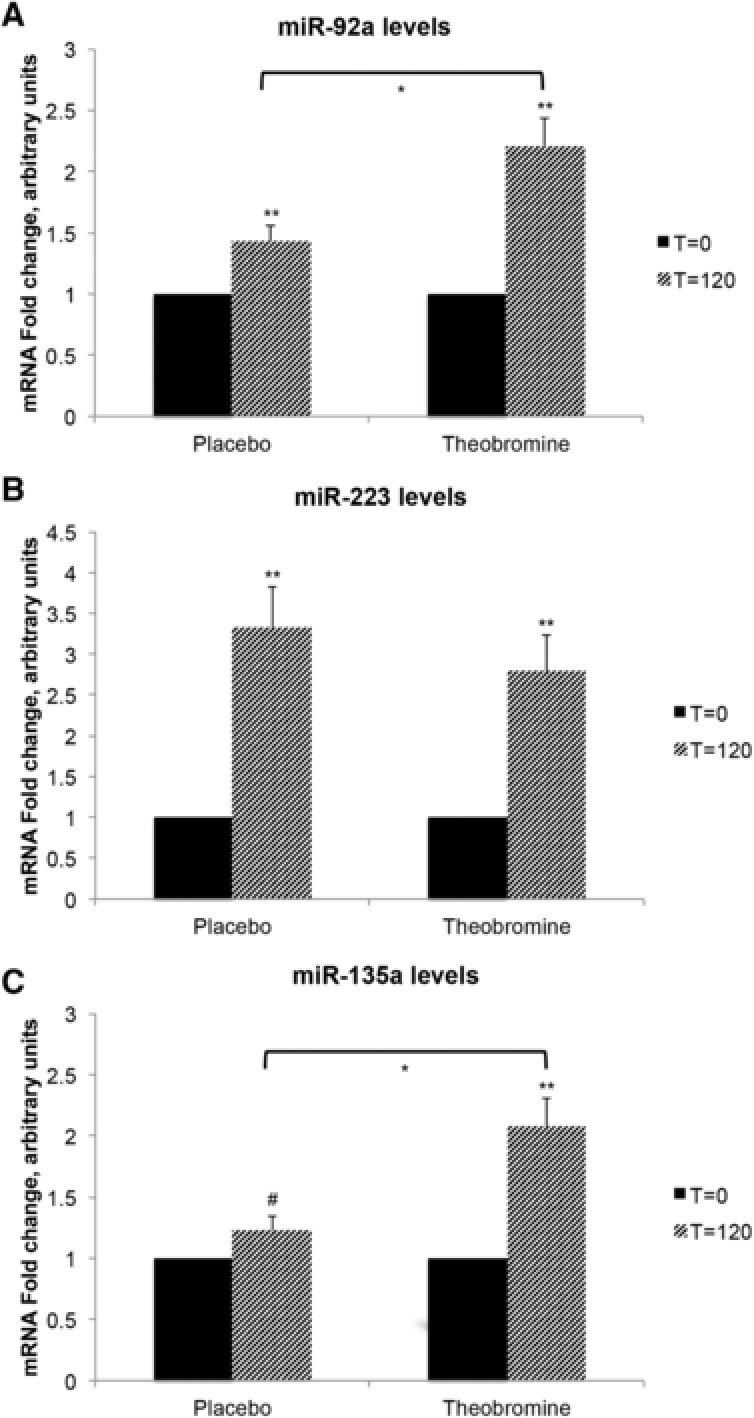
Fold changes of postprandial miRNAs levels 2 h after high‐meal consumption. Postprandial gene expression (*T* = 120) relative to the fasting gene expression (*T* = 0), in the placebo and the theobromine group for: A) miR‐92a: placebo, *n* = 41; theobromine, *n* = 43. B) miR‐223: placebo, *n* = 43; theobromine, *n* = 42. C) miR‐135a: placebo, *n* = 43; theobromine, *n* = 44. Postprandial effect was assessed with one‐samples *t*‐test, with the test value set to 1: *T* = 120 significantly different from *T* = 0; *p ≤ 0.01; **p ≤ 0.001; ^#^ *p* = 0.06. Difference in changes between theobromine and placebo was performed with a paired‐samples *t*‐test: significantly different from change in placebo after meal consumption.

During both periods, that is, with or without theobromine, miR‐92a levels were significantly higher after meal consumption ([+1.21; 95% CI: 0.74, 1.67; *p* < 0.001]; [+0.43; 95% CI: 0.16, 0.69; *p* < 0.01], respectively). MiR‐223 levels were also increased after the meal intake ([+1.79; 95% CI: 0.89, 2.68; *p* < 0.001]; [+2.33; 95% CI: 1.32, 3.35; *p* < 0.001], respectively). Finally, miR‐135a levels were increased during the theobromine period after test meal consumption (+1.08; 95% CI: 0.62, 1.54; *p* < 0.001), while only a trend was observed during the control period (+0.23; 95% CI: −0.01, 0.46; *p* = 0.06). Postprandial changes were significantly higher after the theobromine supplementation for miR‐92a and miR‐135a ([+0.85; 95% CI: 0.34, 1.36; *p* < 0.01]; [+0.78; 95% CI: 0.32, 1.25; *p* < 0.01], respectively), as compared to the placebo period. No difference in the postprandial responses between the two periods was found for miR‐223 (−0.37; 95% CI: −1.71, 0.97; *p* = 0.58) (**Figure** 2).

### 3.3 Correlation between Changes in MiRNAs Levels with Changes in Biochemical Parameters and Cholesterol Efflux Capacity

As miR‐92a levels significantly decreased after 4 weeks of theobromine consumption, correlations between fasting changes in miR‐92a levels and fasting changes in biochemical parameters and cholesterol efflux were calculated. No statistically significant associations were found, which was expected, as the parameters were not changed after the theobromine consumption.

During the placebo period, postprandial changes in miR‐223 levels significantly correlated with age (*r* = −0.43, *p* ≤ 0.01) and with postprandial changes in TAG (*r* = 0.31, *p* < 0.05). No associations between postprandial changes in miR‐92a and miR‐135a with age and any of the changes in the clinical characteristics were observed.

## 4 Discussion

In this study, a daily intake of theobromine for 4 weeks did not affect fasting HDL‐mediated cholesterol efflux capacity. Also, fasting miR‐223 levels, which have been related to cholesterol efflux in vitro,^7^ and miR‐135a levels were unchanged. However, theobromine intake decreased fasting miR‐92a levels. No correlation between changes in miR‐92a and cholesterol efflux was found. In addition, the intake of the high‐fat meal increased postprandial cholesterol efflux capacity, as well as the levels of the three selected miRNAs, that is, miR‐92a, miR‐223, and miR‐135a, but again no correlation between postprandial changes in efflux and miR expression was found.

Cocoa may protect against CVD by affecting HDL metabolism.^15^, ^24^ It is unlikely however that these potential effects are mediated by changing concentrations of HDL cholesterol. First of all, effects of cocoa on HDL cholesterol are not consistent,^10^, ^11^, ^12^ while it is debatable that increasing HDL cholesterol protects against CVD.^13^ We therefore postulated that cocoa may mediate HDL functionality, here defined as cholesterol efflux capacity. To the best of our knowledge, effects of cocoa and cocoa‐rich products such as dark chocolate on cholesterol efflux have never been investigated. However, it has been suggested that flavonoids from cocoa, including flavones, isoflavones, flavanones, flavonols, flavanols, and anthocyanins,^25^ may improve cholesterol efflux capacity in vitro.^26^ Extrapolation of these in vitro findings to the human in vivo situation is difficult, as the doses used in these in vitro studies were high as compared to the estimated dietary flavonoids intake from, for example, the US recommendations,^27^ while bioavailability and bioconversion of these compounds is not taken into account.^26^ In two clinical studies, dietary anthocyanin increased cholesterol efflux capacity from macrophages in dyslipidemic^28^ and hypercholesterolemic^29^ adults. In contrast, consumption of isoflavones for 3 months did not affect SR‐BI^30^ and ABCA1‐mediated cholesterol efflux^31^ in postmenopausal women. Thus, although some studies suggest that certain flavonoids have beneficial effects, our results do not suggest that theobromine consumption affects the ability of HDL to stimulate ABCA1‐mediated cholesterol efflux from macrophages. A relevant question is how the apparent discrepancies between studies can be explained. One possible explanation is that we recently found that a daily intake of 500 mg theobromine slightly, but significantly increased hsCRP concentrations.^19^ As a proinflammatory status is known to lower HDL‐mediated cholesterol efflux,^18^ this increase in hsCRP might have masked a potential beneficial effect of the intervention on cholesterol efflux. However, it should be noted that we observed no correlations between cholesterol efflux and hsCRP at baseline or between the changes in these two parameters. In addition, subjects suffering from metabolic syndrome surprisingly showed an increased ABCA1‐mediated cholesterol efflux.^32^ Although our study population had some characteristics of the metabolic syndrome, they were not suffering from metabolic syndrome. It would therefore also be of interest to test the effect of theobromine in other populations such as people with or without the metabolic syndrome.

Although postprandial dietary effects on cholesterol efflux have not been largely explored, it has been suggested that cholesterol efflux increases during the postprandial phase,^33^ in particular ABCA1‐ and SR‐BI‐mediated efflux.^34^ Moreover, acute walnut oil consumption increased postprandial cholesterol efflux from THP‐1 human monocytes 4 h after the intake, as compared to control.^35^ Comparable results have been reported for the acute consumption of whole walnuts using J774 macrophages.^36^ The fatty acid composition of the meal may be important, as a test meal with a high content of monounsaturated fatty acids increased cholesterol efflux from 4 to 8 h after the intake, while polyunsaturated fatty acid had no effect. Changes in efflux capacity were associated with changes in the phospholipid composition of HDL particles.^37^ We also found a postprandial increase in cholesterol efflux 2 h after the intake of the test meal. However, this effect was not changed by theobromine consumption. As the phospholipid content of HDL is a significant predictor of cholesterol efflux capacity,^38^ the intake of a high‐fat meal may lead to the production of more functional HDL particles, via an enrichment in phospholipids. In addition, preβ‐HDL concentrations have been shown to increase in the postprandial state in overweight and obese subjects.^39^ As preβ‐HDLs are the primary acceptor of cholesterol via the ABCA1 transporter, it is likely that preβ‐HDLs are partly responsible for the postprandial increase in cholesterol efflux.

Effects of theobromine on miR‐92a, miR‐223, and miR‐135a, three miRNAs known to be abundantly present in HDL particles, were investigated in the present study. MiR‐92a expression in serum and plasma was increased in unstable CAD patients, suggesting that this miRNA could serve as biomarker for the detection of CAD.^6^, ^40^ Further, in plasma, miR‐135a levels were decreased in subjects with familial hypercholesterolemia, while miR‐223 levels were increased.^3^ MiR‐223 plays a role in cholesterol homeostasis and increased ABCA1‐mediated cholesterol efflux from hepatocytes.^7^ However, our results do not support this finding in J774 macrophages, which might be due to the fact that a different cell type and acceptor were used. The associations between cholesterol efflux and miR‐92a and miR‐135a have never been investigated, but we did not find any association between miR‐92a and miR‐135a with cholesterol efflux capacity.

Exosomes also carry specific miRNAs.^3^ As exosomes and HDL particles have overlapping densities, the isolation of pure HDL particles is challenging.^41^ MiR‐223^42^ and miR‐92a^43^ are present in exosomes, which might have contributed to the present results. To our knowledge, the presence of miR‐135a in human exosomes has not been examined.

Fasting miR‐223 and miR‐135a levels can be changed by dietary intervention. Four weeks of dietary trans‐fat consumption, from industrial or ruminant origin, increased concentrations of HDL‐miR‐223 and miR‐135a. Variations in miR‐223 levels were associated with a decrease in HDL‐C and an increase in CRP concentrations, while variations in miR‐135a levels were associated with an increase in LDL‐C and TAG concentrations.^44^ In addition, HDL‐miR‐223 levels were reduced after 12 weeks weight loss using a high‐protein diet, independent of HDL composition and size.^45^ In our present study, we found no effect of theobromine on miR‐135a and miR‐223, but fasting miR‐92a levels were decreased. Although it is tempting to speculate about positive effects related to this effect since miR‐92a might be involved in angiogenesis and atherosclerosis,^6^, ^40^, ^46^, ^47^ longer‐term intervention studies specifically targeting miR‐92a are needed before it can be concluded whether these effects of theobromine are beneficial or not. However, when found protective, changes in miR‐92a levels are not associated with HDL‐efflux capacity, apoA‐I and TAG concentrations, as they did not change after the 4‐week theobromine consumption. A slight increase in HDL‐C concentrations was found after the theobromine supplementation, but this was also not associated with changes in miR‐92a. Changes in HDL particles size and/or composition could be a possible explanation. However, we have recently reported that theobromine consumption for 4 weeks was not associated with changes in the cholesterol concentrations in total HDL particles, as well as in any of the HDL subfractions.^48^

This is the first time that the postprandial responses of miR‐92a, miR‐223, and miR‐135a have been investigated. Following meal intake, both miR‐92a and miR‐223 levels were significantly increased, while those of miR‐135a tended to increase. Postprandial changes in miR‐92a and miR‐135a were significantly higher after the 4‐week theobromine consumption. These findings are difficult to explain and were not related to postprandial changes in concentrations of HDL‐C, apoA‐I and TAG, or cholesterol efflux, which did not change. Not much is known regarding changes of miRNAs levels during the postprandial phase. A study performed in rats investigated the effect of proanthocyanidin consumption on miRNAs levels, and found a decreased in miR‐122 and miR‐33a levels in the liver 1 h after intake.^49^ In rainbow trout, levels of miRNAs involved in insulin signaling were increased 4 h after meal intake.^50^ However, no clear explanations for these postprandial changes were given but it was suggested that it might be attributed to changes in nutrients composition or to variation in hormonal factors. Indeed, the postprandial increase in miRNAs levels might be related to the normal increase in insulin observed after meal intake, as our selected miRNAs have been shown to be involved in the regulation of insulin biosynthesis and signaling pathway.^51^, ^52^ In addition, miRNAs are found in food products, and can be absorbed.^53^, ^54^, ^55^, ^56^ However, if the specific miRNAs studied in the present study are associated with food products and theobromine is not known. In addition, if the postprandial increases in miRNAs levels are due to absorption from dietary sources or to an increase in the expression of genes coding for miRNAs needs further investigations. In this study, a high‐fat meal was used for the postprandial test. It would be of interest to investigate if the same postprandial effects on miRNAs levels depend on macronutrient composition.

In conclusion, the present study shows that high‐fat meal intake increases postprandial cholesterol efflux capacity, as well as miRNAs levels. However, 4 weeks of theobromine consumption did not change HDL‐mediated cholesterol efflux capacity at baseline and postprandially. In addition, theobromine could exert anti‐atherogenic properties by reducing miR‐92a levels. We cannot exclude that miRNAs carried by exosomes might have contributed to the present results.

## Conflict of Interest

The authors declare no conflict of interest.

## Supporting information

Supporting Information Table S1: Baseline characteristics of the study population
Supporting Information Table S2: Composition of the test drinks (20ml)
