# Supplementary material for: Theobromine Does Not Affect Fasting and Postprandial HDL Cholesterol Efflux Capacity, While It Decreases Fasting miR‐92a Levels in Humans
Source: Mol Nutr Food Res. 2018 Jun 19;62(13):1800027. doi: 10.1002/mnfr.201800027 (PMC6055688; doi:10.1002/mnfr.201800027)
Supplement: Supplementary file 1 — Supporting Information Table S1: Baseline characteristics of the study population Supporting Information Table S2: Composition of the test drinks (20ml) [file MNFR-62-na-s001.docx]

Supporting Information Table S1: Baseline characteristics of the study population

|  | Baseline (n=44) | | |
| --- | --- | --- | --- |
| Age (years) | 60.3 | ± | 5.5 |
| BMI (kg/m²) | 29.2 | ± | 3.0 |
| Total cholesterol (mmol/L) | 5.60 | ± | 1.03 |
| TAG (mmol/L) | 1.75 | ± | 0.73 |
| LDL-C (mmol/L) | 3.72 | ± | 0.87 |
| HDL-C (mmol/L) | 1.08 | ± | 0.19 |
| apoA1 (g/L) | 1.37 | ± | 0.19 |
| apoB (g/L) | 1.23 | ± | 0.25 |
| Glucose (mmol/L) | 6.02 | ± | 0.71 |
| Insulin (uU/mL) | 15.13 | ± | 6.10 |
| HOMA-IR | 2.02 | ± | 0.80 |
| CRP (mg/L) | 3.09 | ± | 2.95 |
| SBP (mmHg) | 130 | ± | 14 |
| DBP (mmHg) | 85 | ± | 8 |
| Heart Rate (bpm) | 68 | ± | 11 |

Values are means ± SD

TAG: triacylglycerol; LDL-C: low density lipoprotein cholesterol; HDL-C: high density lipoprotein cholesterol; apoB: apolipoprotein B; apoA1: apolipoprotein A1; HOMA-IR: model assessment-estimated insulin resistance; CRP: C-reactive protein; SBP: systolic blood pressure: DBP: diastolic blood pressure; bpm: beats per minute

Supporting Information Table S2: Composition of the test drinks (20ml)

|  | Placebo | Theobromine |
| --- | --- | --- |
| Theobromine (mg) | - | 500 |
| Microcrystalline cellulose (mg) | 500 | - |
| Methyl cellulose (mg) | 150 | 150 |
| Sucralose (mg) | 10 | 10 |
| Sodium benzoate (mg) | 100 | 100 |
| Anise 0.1% (mg) | 20 | 20 |
| Water | Until 20 g | Until 20g |
